# Association of mtDNA M/N haplogroups with systemic lupus erythematosus: a case-control study of Han Chinese women

**DOI:** 10.1038/srep10817

**Published:** 2015-06-03

**Authors:** Youzhou Tang, Li Wang, Min Zhu, Ming Yang, Kuangbiao Zhong, Qing Du, Hao Zhang, Ming Gui

**Affiliations:** 1 Department of Nephropathy and Rheumatology, The third Xiangya Hospital of Central South University, No.138 Tongzipo Road, Changsha, 410013, China; 2 Department of Blood Transfusion, The fourth Central Hospital of Tianjin, No.1 Zhongshan Road, Tianjin, 300000, China; 3 School of life sciences, Central South University, No.172 Tongzipo Road, Changsha, 410013, China; 4 Department of Urology, The third Xiangya Hospital of Central South University, No.138 Tongzipo Road, Changsha, 410013, China

## Abstract

To investigate whether mitochondrial DNA haplogroups M or N are related to occurrence or manifestations of systemic lupus erythematosus (SLE), we collected M/N haplogrouping and clinical characteristics from 868 Han Chinese women with SLE, as well as for 870 age-matched healthy Han Chinese control women. M/N haplogroups were determined in all subjects using allele-specific amplification. The frequency of M haplogroup in all patients was 429 (49.4%) and the frequency of N haplogroup, 439 (50.6%). The corresponding frequencies in controls were 456 (52.4%) and 414 (47.6%) (P = 0.213). Among women older than 50 years at onset age, the N haplogroup was significantly higher in patients than in healthy controls (59.6% vs 41.7%, P = 0.042). The N haplogroup was associated with significantly higher risk for certain SLE characteristics: hematological system damage (OR 2.128, 95%CI 1.610 to 2.813), skin impairment (OR 1.873, 95%CI 1.428 to 2.457), neurological disturbance (OR 3.956, 95%CI 1.874 to 8.352) and alopecia (OR 1.322, 95%CI 1.007 to 1.737 ). Our results suggest that in Han Chinese women, the mtDNA N haplogroup is associated with higher risk of late-onset SLE, skin impairment, neurological disturbance, hematological system damage and alopecia.

Polymorphisms in mitochondrial DNA (mtDNA), a 16.6-kbp double-stranded molecule encoding several rRNAs, tRNAs and proteins^1^, have been linked to risk of several complex diseases, including diabetes mellitus, cancer, and Parkinson’s^2^. In particular, when multiple single-nucleotide polymorphisms (SNPs) occur together as part of an mtDNA haplotype, oxidative respiratory chain activity can be affected, disturbing the ratio of production of ATP to reactive oxygen species (ROS). If ATP production becomes insufficient, abnormal apoptosis can result^3^,^4^; if ROS production becomes excessive, the cells are unable to repair the oxidative stress, leading to permanent DNA damage. Consequently, changes in the ratio of ATP to ROS production can influence cellular proliferation and activation of programmed cell death.

DNA damage and T cell pathology due to excessive ROS production occur in systemic lupus erythematosus (SLE)^5^,^6^,^7^, a complex autoimmune disease that can damage multiple human systems and organs. A genome-wide association study (GWAS) of SLE has identified more than 40 nuclear genes that may predispose individuals to SLE^8^. Numerous studies further suggest that the disease arises through the effects of multiple genes as well as through gene-environment interactions. These factors help to explain why 87.5%-90% SLE patients are female, and why Han Chinese show much higher incidence of the disease, earlier age of onset and higher risk of kidney involvement than Europeans^9^,^10^,^11^.

Ethnic and regional genetic heterogeneity may reduce the reliability of SLE studies based on SNPs, including GWAS. This is similar to the case in diabetes research, where GWAS have reported conflicting results for the same disease susceptibility gene candidates^12^,^13^.

Genetic studies of disease susceptibility may be more reproducible and accurate when based on not only individual SNPs but also haplotypes, since human genetic studies focusing on haplotypes indicate that several SNPs can show linkage, giving rise to synthetic genetic effects. Given a US study suggesting that specific mtDNA SNP haplogroups may affect risk of breast cancer in women^3^, also, researchers have already focused genetic factors relating SLE not only on SNP but also on SNP linkage haplotypes like Toll-like receptors^14^. We wondered whether mtDNA haplogroups may be associated with risk of SLE or particular SLE characteristics. Here we explore possible correlations between mtDNA SNP haplogroups in Han Chinese and risk of SLE, as well as risk of particular SLE characteristics. We focused on mtDNA SNPs 8701 (A/G), 10398 (A/G) and 10400 (C/T), which are thought to explain most SNP variation between Europeans and Han Chinese. Most Europeans carry sequences for these and other SNPs that are collectively denominated as haplogroup N, while Han Chinese carry haplogroups N and M [searching from mtDNA database].

## Results

### Characteristics of patients and controls

The control group included 870 healthy women with an average age of 34.01 ± 10.28 years; the patient group, 868 women with an average age of 35.42 ± 11.66 years. The age distribution was similar between the two groups (P > 0.05).

Age of SLE onset in patients averaged 31.85 ± 10.69 years. Most frequent clinical manifestations at onset included hematological system damage, renal impairment, arthritis, skin lesion and positivity for anti-dsDNA antibodies (Table 1).

### M/N haplotyping of mtDNA

**Figure 1:**
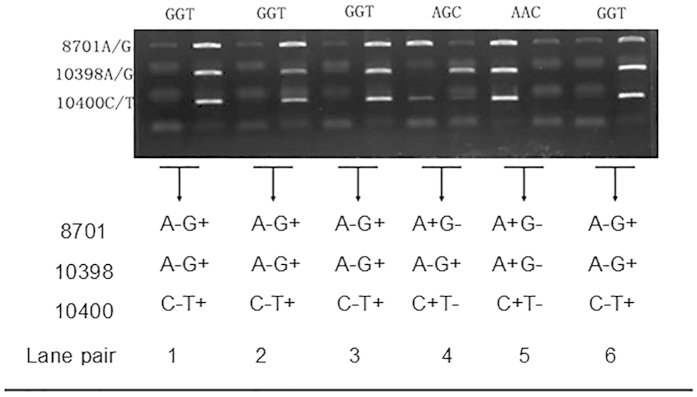
Representative results of allele-specific amplification to haplogroup Han Chinese women with SLE and healthy Han Chinese controls. Reactions were analyzed by 1.2% agarose gel electrophoresis. Amplification reactions for each SNP were loaded across two adjacent lanes. The position of each SNP is labeled at left. For example, in lane pair 1, positive bands were observed for 8701 G, 10398 G and 10400 T. A sequence of GGT at the 3 SNPs was classified as an mtDNA M haplogroup, while sequences of AGC and AAC were classified as an mtDNA N haplogroup.

Haplogroup was determined in all patients and controls, the way to determine haplogroup M and N are showed in Fig. 1. Frequencies of all three mtDNA SNPs 8701 (A/G), 10398 (A/G) and 10400 (C/T) showed Hardy-Weinberg equilibrium in both groups (P > 0.05). The M haplogroup occurred in 49.4% of patients and the N haplogroup in 50.6%. The corresponding frequencies in the control group were 52.4% and 47.6% (P = 0.213).

To control for possible confounding effects due to differences in estrogen, which is important in SLE pathogenesis observed by many observations, we defined subgroups of patients and controls according to whether they were younger or older than 50 years at onset age, which corresponds to the average age of menopause in Han Chinese. The results suggest that among women younger than 50 years, haplogroup frequencies were not significantly different between patients and controls (P = 0.861; Table 2). However, among women older than 50 years, the mtDNA N haplogroup occurred significantly more frequently among SLE patients than among healthy controls (OR 2.070, 95%CI 1.021 to 4.196). This suggests that the mtDNA N haplogroup may be a risk factor for late-onset SLE.

Since all subjects in this study were older than 14 years old, we did not consider estrogen level before menarche.

### Association between mtDNA haplogroups M/N and clinical manifestations of SLE

Certain genetic polymorphisms of SLE show strong associations with age of onset of SLE(<30 yrs old) and with some complications of SLE^15^,^16^. Therefore, we analyzed our data for such possible associations. The mtDNA N haplogroup showed a significant association with the following SLE complications: hematological system damage, skin impairment, neurological disturbance and alopecia(Table 3). On the other hand, neither mtDNA haplogroup was significantly associated with 30 years age of onset, kidney damage, positivity for anti-dsDNA antibodies, arthritis, photosensitivity, oral ulcers, or occurrence of Raynaud’s phenomenon(Table 3).

### Association between mtDNA M/N haplogroup and SLE activity

We analyzed our data for possible associations between M/N haplogroup and SLE activity based on SLEDAI score. The results showed that the N haplogroup was associated with significantly higher SLE activity than the M haplogroup (Table 4).

## Discussion

We show here for the first time that there is an association between mtDNA M/N haplogroups and risk of SLE in Han Chinese women. While neither haplogroup is associated with increased risk in women younger than 50 years at onset age, the N haplogroup is associated with significantly higher SLE risk in women older than 50 at onset age. This is surprising given the previously proposed rule that nuclear genes contribute more to risk of SLE in younger individuals, whereas environmental factors contribute more to risk in older individuals^17^. One way to reconcile our finding with this general rule is that the N haplogroup interacts with environmental factors and thereby becomes a risk factor for SLE only after interacting with the environment for a sufficiently long time.

SNPs in mtDNA have already been shown to interact with the environment. Mutation rates of mtDNA are higher in skin tissue exposed to ultraviolet irradiation and lung tissue exposed to cigarette smoke than in unexposed tissues. This makes mtDNA mutations and resulting changes in ROS production a sensor of exposure to environmental changes^18^.

The association between the N haplogroup and risk of SLE in older women likely reflects the combined action of several mtDNA SNPs. The SNP 8701 A/G is a mutation-prone site in the gene encoding the ATP6 subunit of ATP synthase^19^, and the mutation 8701 A→G changes a lysine into threonine, reducing ATP production and inducing abnormal apoptosis^20^. The mutation 10398 G→A changes a threonine into lysine in the ND3 subunit of mitochondrial complex I, which increases ROS production, and the resulting oxidative stress may disturb the immune response and cause inflammation^21^.

Our data suggest that, the mtDNA haplogroup is associated with significantly increased risk of several clinical manifestations of SLE: neurological disturbance, hematological system damage and alopecia. Associations of mtDNA SNPs with neurological disturbances and hematological system damage have also been reported in other diseases including stroke and epilepsy^2^. It is unclear why the N haplogroup, associated with reduced ATP production, shows an association with these types of complications. We hypothesize that nervous tissue, blood cells, and the germinal layer of hair follicles have a higher metabolism and are more sensitive to ATP insufficiency. Future studies should examine this possibility in detail.

We found the mtDNA N haplogroup to be associated with higher SLE activity. We speculate that at least one reason for this association is that the decrease in ATP production and increase in ROS production in mitochondria of T lymphocytes induces apoptosis and necrosis^6^,^7^. Necrosis in T lymphocytes in turn promotes SLE occurrence and progression because the lymphocytes release high mobility group box-1 protein and heat shock protein 70/90; they also stimulate monocytes to release TNF-α and IL-1 and differentiate into dendritic cells, which release large amounts of IFN-α^22^. Future studies should explore possible mechanisms whereby the mtDNA N haplogroup leads to higher SLE activity.

The central role of mtDNA in vital metabolic processes and physiological reactions means that its polymorphisms are unlikely to cause grave effects for carriers, since such mutations would likely be lethal. Consistent with this idea, our data suggest that the mtDNA N haplogroup acts as a minor predisposing genetic factor in SLE.

## Material and Methods

The study protocol was approved by the Ethics Committee of the third Xiangya Hospital, Central South University, Changsha, China. All participants provided written informed consent according to the Declaration of Helsinki prior to the study. All methods were carried out in accordance with the approved guidelines.

### Subjects

Han Chinese women with SLE were recruited between October 2012 and June 2013 from among outpatients and inpatients in the rheumatology departments of Xiangya Hospital, the Second Xiangya Hospital and the Third Xiangya Hospital, all affiliated with Central South University. All patients satisfied the criteria for SLE published by the American College of Rheumatology in 1997. Patients who also had other connective tissue diseases, severe infection or a family history of autoimmune diseases were excluded.

During patient recruitment, healthy Han Chinese women were also recruited as controls. Women were recruited after coming in for voluntary health check-ups at the community health center of the Third Xiangya Hospital. Controls had no history of SLE on their first, second or third-degree relatives.

### Clinical characteristics of patients with SLE

The following clinical information was collected: patient ethnicity, gender, current age, age at disease onset, disease stage, family history, clinical manifestations, antinuclear antibody spectrum, anti-dsDNA antibody status, white blood cell count, platelet count, urine protein profile, 24-hr urine protein quantitation, urine red blood cell count and renal cast status. SLE activity was evaluated using the scoring system of the SLE disease activity index (SLEDAI). The following ratings were used: 1, severe (SLEDAI ≧ 15 points); 2, moderate (SLEDAI = 10–14); 3, mild (SLEDAI = 5–9); 4, mildly inactive (SLEDAI = 0–4).

### DNA extraction

DNA was extracted from peripheral white blood cells using conventional methods. The quality of genomic DNA was verified using agarose gel electrophoresis, and concentration was determined from the absorbance at 260 nm. Genomic DNA was stored at −80 ^o^C.

### M/N haplotyping

The following SNPs were initially considered for M/N haplotyping of mtDNA from our patients and controls (www.mitomap.org): 8701 (*ATP6*), 10398 (*ND3*), 10400 (*ND3*), 9540 (*COII*), 14783 (*cytb*), 15043 (*cytb*) and 15301 (*cytb*). Of these SNPs, 8701 (A/G) and 10398 (A/G) are missense mutations, and both are in tight linkage with 10400 (C/T).

Therefore the mtDNA SNPs 8701 (A/G), 10398 (A/G) and 10400 (C/T) were screened to determine M/N haplogroup. Individuals with the sequence GGT for these loci were haplogrouped as M, while those with AAC or AGC were haplogrouped as N(Illustrations are showed in Fig. 1).

Haplotyping was performed using allele-specific amplification and the following primers:

1. 8701 F, 5`-AAGATTAAGAGAACCAACACCTCT-3`;
2. 8701NA, 5`-CGTCCTTTAGTGTTGTGTATCGT-3`;
3. 8701NG, 5`-CGTCCTTTAGTGTTGTGTATCGC-3`;
4. 26 R, CTAGTATGGCAATAGGCACAATA;
5. 10398NA, GACTACAAAAAGGATTAGACTCAA;
6. 10398NG, GACTACAAAAAGGATTAGACTCAG;
7. 10400FC, 5`-CTACAAAAAGGATTAGACTGTACC-3`;
8. 10400FT, 5`-CTACAAAAAGGATTAGACTGTGCT-3`.

Amplifications (10 μL) contained 60 ng DNA, 20 mM Tris-HCl (pH 8.8), 10 mM KCl, 10 mM (NH4) _2_SO_4_, 1.5 mM MgCl_2_, 0.1% Triton X-100, 0.2 μM each primer, 0.2 mM each dNTP, and 0.2 U Taq DNA polymerase (Promega, USA). Cycling conditions were 94 °C for 5 min and 35 cycles of 94 °C for 30 s, 57.5 °C for 28 s, and 72 °C for 30 s.

### Statistical analysis

The χ^2^ test was used to determine whether the relative proportions of mtDNA M/N haplogroups were in Hardy-Weinberg equilibrium in SLE patients and healthy controls. Possible associations between M/N haplogroup and age of SLE onset or occurrence of specific SLE symptoms and ages were assessed using the χ^2^ test or Fisher’s exact test. Possible associations between M/N haplogroup and SLE activity were assessed using the Mann-Whitney U rank sum test. All statistical tests were performed in SPSS 18.0 (IBM, Chicago, USA), and P < 0.05 was defined as the threshold of significance. In Table 2, in order to exclude estrogen’s effect on SLE, we made a priori to subgroup the population into ages above or below 50 yrs old at onset age.

## Additional Information

**How to cite this article**: Tang, Y. *et al*. Association of mtDNA M/N haplogroups with systemic lupus erythematosus: a case-control study of Han Chinese women. *Sci. Rep.* **5**, 10817; doi: 10.1038/srep10817 (2015).

## Figures and Tables

**Table 1:** Clinical manifestations of Han Chinese women with SLE.

**Manifestation**	**n**	**%**
Hematological system damage	538	62
Renal impairment	520	60
Arthritis	503	58
Skin impairment	486	56
Positivity for anti-dsDNA antibodies	456	52.5
Alopecia	347	40
Photosensitivity	139	16
Oral ulcer	87	10
Nervous system change	43	5
Raynaud phenomenon	42	4.8

**Table 2:** Frequencies of mtDNA haplogroups in Han Chinese patients with SLE and healthy controls.

**Group or subgroup**	**mtDNA haplogroup, n (%)**		**P**	**OR**	**95%CI**
	**M**	**N**			
Patients with SLE	429 (49.4)	439 (50.6)	0.213*		
<50 years old at onset age	406 (50.1)	405 (49.9)	0.861#		
≧50 years old at onset age	23 (40.4)	34 (59.6)	0.042^Δ^	2.070	1.021 to 4.196
Healthy controls	456 (52.4)	414 (47.6)			
<50 years old	396 (49.6)	402 (50.4)			
≧50 years old	42 (58.3)	30 (41.7)			

^*^between all patients and controls.

^#^between patients younger than 50 years at onset age and controls younger than 50 years.

^Δ^between patients 50 years or older at onset age and controls 50 years or older.

**Table 3:** Frequencies of mtDNA M/N haplogroups in Han Chinese patients with SLE and specific clinical manifestations of the disease

**Manifestation**	**mtDNA M/N haplogroup frequency, n (%)**				**χ**^**2**^	**P**	**OR**#	**95% CI**
	**Patients with manifestation**		**Patients without manifestation**					
	**N**(%)	**M**(%)	**N**(%)	**M**(%)				
Onset age < 30 years	203 (49.8)	205 (50.2)	236 (51.3)	224 (48.7)	0.208	0.649	0.94	0.72 to 1.227
Lupus nephropathy	260 (50.0)	260 (50.0)	169 (48.6)	179 (51.4)	0.172	0.678	1.059	0.807 to 1.390
anti-dsDNA ( + )	225 (49.3)	231 (50.7)	203 (49.3)	209 (50.7)	0.000	0.984	1.003	0.768 to 1.309
Hematological system damage	344 (63.9)	194 (36.1)	150 (45.5)	180 (54.5)	28.504	< 0.001*	2.128	1.610 to 2.813
Arthritis	236 (46.9)	267 (53.1)	193 (52.9)	172 (47.1)	3.004	0.083	0.788	0.601 to 1.032
Skin impairment	293 (60.3)	193 (39.7)	171 (44.8)	211 (55.2)	20.716	< 0.001*	1.873	1.428 to 2.457
Alopecia	192 (55.3)	155 (44.7)	252 (48.3)	269 (51.7)	4.041	0.044*	1.322	1.007 to 1.737
Photosensitivity	79 (57.1)	60 (42.9)	359 (49.2)	370 (50.8)	2.690	0.101	1.357	0.941 to 1.956
Oral ulcer	35 (40.2)	52 (59.8)	387 (49.6)	394 (50.4)	2.723	0.099	0.685	0.437 to 1.076
Nervous system disturbances	34 (79.1)	9 (20.9)	403 (48.8)	422 (51.2)	14.932	< 0.001*	3.956	1.874 to 8.352
Raynaud phenomenon	18 (42.9)	24 (57.1)	411 (49.8)	415 (50.2)	0.761	0.383	0.757	0.405 to 1.416

^*^P < 0.05.

^#^ORs refer to the risk associated with the mtDNA N haplogroup relative to the mtDNA M haplogroup.

**Table 4:** Frequencies of mtDNA M/N haplogroups in Han Chinese patients with different SLE activity levels.

SLE activity*	**mtDNA M/N haplogroup frequency, n (%)**	
	**M**	**N**
No. of subjects	429	439
1	49 (11.4)	63 (14.4)
2	170 (39.6)	236 (53.8)
3	154 (36.0)	112 (25.5)
4	56 (13.0)	28 (6.30)
Rank mean	473.24	396.65
Z	−4.836	
*P*	<0.001	

^*^Based on SLE disease activity index (see Methods).
